# MONILETHRIX IN THREE GENERATIONS

**DOI:** 10.4103/0019-5154.41660

**Published:** 2008

**Authors:** Gurcharan Singh, K Siddalingappa, K C Nischal, L Chandra Naik, K Lokanatha

**Affiliations:** *From the Department Skin and STD, SDUMC, Kolar, India*; 1 *From the Department of Skin and STD, VIMS, Bellary, India*; 2 *From the Department of Skin and STD, AIMS, Bellur, Karnataka, India. E-mail: naanu0006@yahoo.co.in*

Monilethrix is a rare autosomal dominant hair shaft disorder characterized by uniform elliptical nodes and intermittent constrictions that result in hair fragility and patchy or diffuse alopecia. We report here a case of monilethrix in a healthy four year-old female child with a family history of a similar condition in the patient's mother and maternal grandmother.

Monilethrix, a rare hair shaft disorder, was first described by Smith in 1879 as a rare nodose condition of the hair and the term “monilethrix” was later coined by Radcliff Crocker.^1^,^2^ Inheritance is usually autosomal dominant although a recessive mode has also been reported. The affected persons tend to have short, sparse, dry, fragile beaded hair which rarely attains lengths of >2 cm, although it has been reported to grow up to 7 cm.^2^ We report here a rare case of this hair shaft disorder occurring in three consecutive generations.

**Fig. 1:**
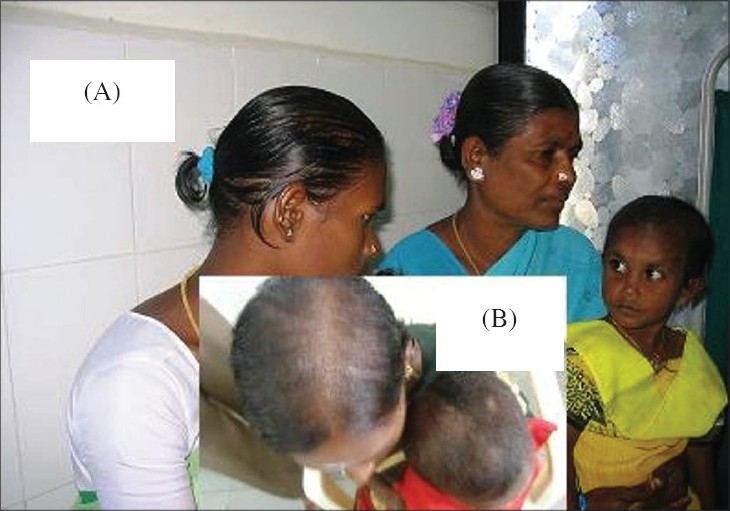
(A) Short, sparse scalp hair of the affected child, mother and grandmother, (B) Short, sparse, fragile scalp hair, predominantly over the vertex of the affected child and mother

**Fig. 2:**
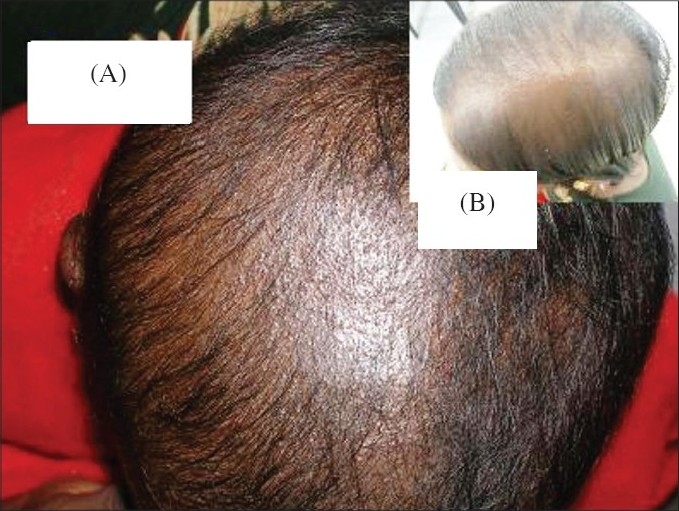
(A) and (B) Follicular papules of the vertex in the affected child and mother, respectively

A four year-old female child of a nonconsanguineous marriage presented to our out-patient department (OPD) with complaints of sparseness and inability to grow long hair over the scalp since birth. It was stated that the hair broke when it reached a certain length. A history of similar affliction was present in the child's mother and maternal grandmother, and both of them were reported to have improved gradually with age (Fig. 1). On cutaneous examination, diffuse thinning of scalp hair predominantly over the vertex, was observed. Hair was short, sparse and dry. Multiple small, skin-colored, follicular-oriented papules were present on the surface of the scalp and neck (Fig. 2). Hair in other body sites was normal. The child was of normal height and weight. Dental, ENT (Ear, Nose and Throat), ophthalmic and other systemic examinations revealed no abnormalities. Nails and mucous membranes were normal.

**Fig. 3:**
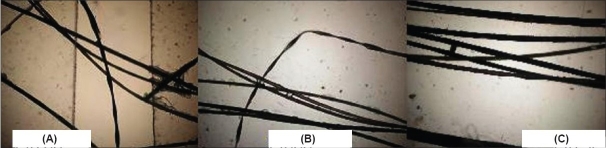
(A), (B), and (C) Microscopic view of the beaded appearance of hair shafts of the affected child, mother and grandmother, respectively

Laboratory investigations: complete hemogram, blood sugar, blood urea, serum creatinine, liver function and routine urine tests were normal. Microscopic examination of the hair showed a characteristic beaded appearance at about 1 mm intervals (Fig. 3), suggestive of monilethrix.

Monilethrix is a rare, inheritable hair shaft disorder. At birth, the hair shaft appears normal but soon thereafter, probably related in part to external trauma, nodes begin to form along the shafts at regular intervals of 0.7-1 mm. Recent studies have demonstrated the abnormalities of cuticle, cortex and keratinizing zones of hair follicles.^3^ The beading or moniliform appearance of the hair shaft is caused by the nodes and internodal thinning of the shaft. The nodes seem to represent normal growth; the internodes are characterized by the wrinkling of corticle cells leading to fragility of the hair, with an absence of medulla.^4^ Degeneration, cytoplasmic vacuolation and abnormal tonofibrils have been observed in the cortical cells with invagination of the cuticle cells into the cortex. The cortical cells are particularly affected in the hair matrix.^5^ The hair on the nape of the neck and occipital region are the most commonly affected parts but hair over the entire scalp and in other body sites such as eyelashes, eyebrows, axillary and pubic hair can also be involved.^6^ Inheritance is usually autosomal dominant with a high penetrance and variable expressivity; however, autosomal recessive and sporadic cases have also been reported.^2^ The disease develops because of mutations in genes (chromosome 12q13) that code Khb1, Khb3 and Khb6, the basic hair keratins in humans. Lanugo hair is normal in the neonatal period. Clinical signs appear when terminal hair characteristics begin to form.^7^ Apart from short, sparse, fragile, nongrowing hair, affected patients may have keratosis pilaris, koilonychia and rarely, systemic disturbances such as mental and physical retardation, syndactyly, cataract, teeth and nail anomalies.^7^

Light microscopy is usually diagnostic and showed typical beaded or moniliform appearance of the hair. An improvement during adolescence and pregnancy has been documented, suggesting a hormonal influence.^6^ There is no specific treatment for monilethrix although improvement has been reported with oral steroids, retinoids, griseofulvin and topical minoxidil. Some cases show improvement with age.^6^,^7^

In our case, the pattern of disease affliction shows autosomal dominant inheritance running in three consecutive generations and gradual improvement of the condition with age.
